# Management of Radicular Cysts Through Enucleation With Curcuma Oral Gel and Absorbable Gelatin Sponge Application

**DOI:** 10.7759/cureus.84297

**Published:** 2025-05-17

**Authors:** Venkata Krishna Irukulla, Godvine Sarepally, Kavya Rachana Chatharasi, John Jims Veeravalli, Ravali Sriram

**Affiliations:** 1 Oral and Maxillofacial Surgery, Panineeya Institute of Dental Sciences and Research Centre, Hyderabad, IND

**Keywords:** curcumin gel, enucleation, gel foam, healing, radicular cyst

## Abstract

Radicular cysts are common pathological entities in oral and maxillofacial surgery, often necessitating surgical intervention for resolution. This case report outlines the successful management of a radicular cyst through enucleation, supplemented by the application of curcumin gel and gel foam. Enucleation of the cyst was meticulously performed, followed by the application of curcuma oral gel curenext® (Abbott Healthcare Pvt. Ltd., Thane, India), which was recognized for its anti-inflammatory properties. Absorbable gelatin sponge Abgel (Healthium Medtech Pvt. Ltd., Mumbai, India) was used to ensure controlled delivery of the curcumin gel. This report exemplifies a promising approach to the treatment of radicular cysts, showcasing the potential benefits of incorporating adjunctive therapies like curcumin gel and gel foam.

## Introduction

Radicular cysts, which emerge from epithelial residues in the periodontal ligament due to periapical periodontitis resulting from pulp necrosis, typically remain asymptomatic and go unnoticed until they are identified during routine periapical radiography [1]. Radicular cysts appear as radiolucent lesions with well-defined, corticated borders on radiographs. In uncommon instances, they may grow larger than 1 cm and cause expansion of the buccal or lingual cortical plates, potentially resulting in thinning of the surrounding bone near the affected tooth [2].

Approximately 52% to 68% of jaw cysts in humans are constituted by these cysts [3]. The highest incidence of these cysts occurs in the third and fourth decades of life, with a prevalence of males [4], a male-to-female ratio of approximately 1.35:1 [5]. Anatomically, periapical cysts can be found in all tooth-bearing areas of the jaw, but they are more commonly observed in the maxillary region than in the mandibular region [3,4,6]. Spontaneous healing of radicular cysts can occur following endodontic treatment or extraction. Nevertheless, certain authors suggest complete surgical enucleation of suspected radicular cysts to ensure the removal of all epithelial remnants [7]. Radicular cysts can present a range of treatment challenges in the field of dentistry. While some smaller radicular cysts can be effectively managed through root canal treatment, others may require surgical intervention for resolution. The choice between these two approaches depends on several factors, including the size and location of the cyst and its impact on surrounding structures.

Over the past few decades, many studies have explored the use of various materials, such as autogenous, allogenous, xenografts, and synthetic grafts, for filling jaw defects. These materials help reduce infections and healing problems, speed up bone regeneration, prevent soft tissue from collapsing into the defect, and improve bone strength [8]. Natural compounds such as curcumin gel, due to their anti-inflammatory and antimicrobial properties, have been explored for their potential in promoting healing. This case report presents the successful surgical management of a large infected radicular cyst using curcumin gel and absorbable gel foam.

## Case presentation

A 45-year-old male patient visited the Department of Oral and Maxillofacial Surgery of Panineeya Mahavidyalaya Institute of Dental Sciences and Research Centre, Hyderabad, with a chief complaint of pain and discharge of pus from the upper front tooth region for one month. The patient has a history of trauma to the upper anterior teeth, which was 20 years ago.

**Figure 1:**
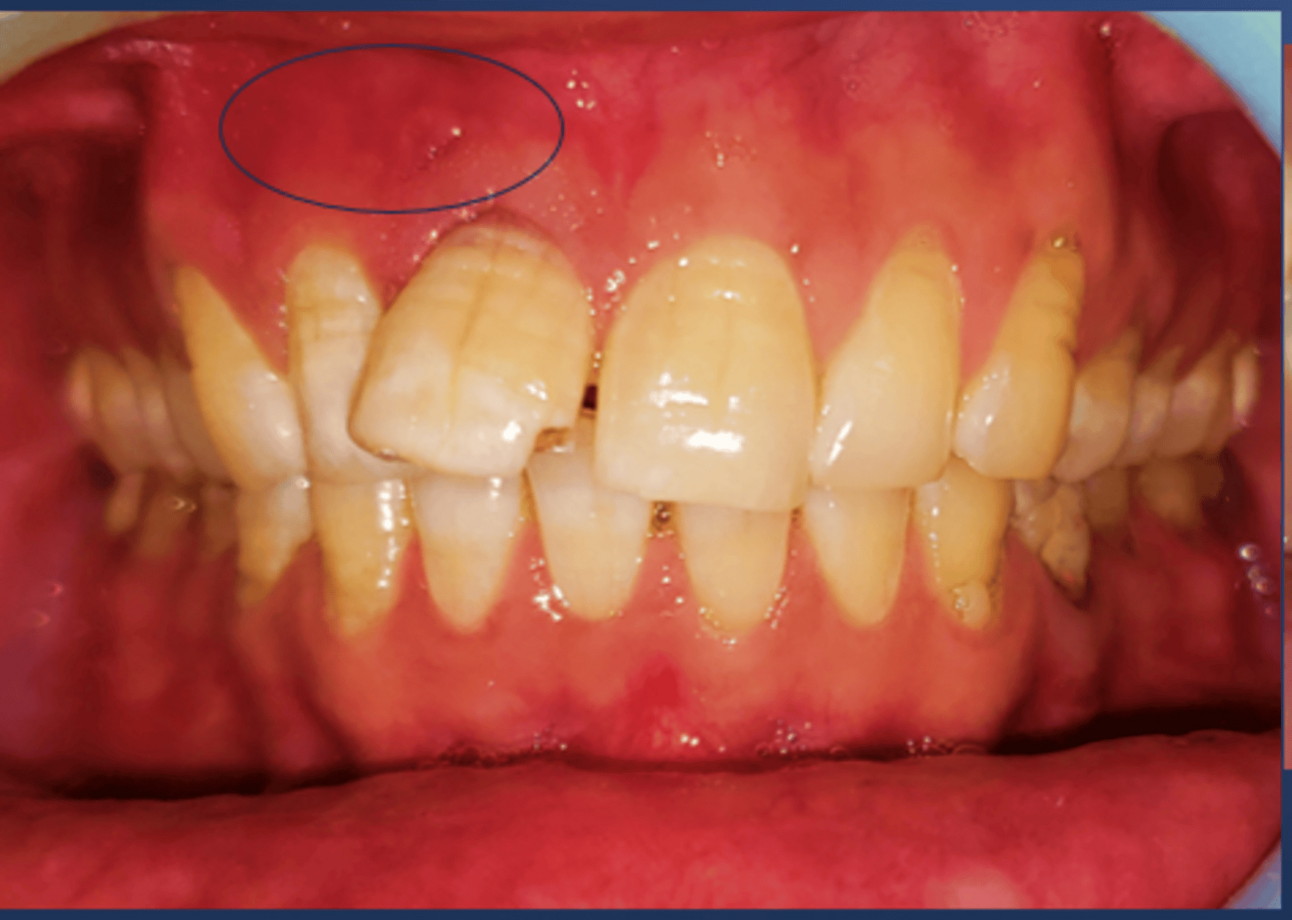
Intra oral image showing erythematous overlying labial mucosa

On intraoral examination, the overlying labial mucosa was found erythematous (Figure 1), extending from 11 to 13 with a sinus tract opening in relation to 11 and tender on palpation. Electric and thermal pulp vitality testing showed negative responses in 11 and 12, while adjacent teeth showed normal responses. Based on the patient’s history of trauma and thorough clinical examination, a provisional diagnosis of infected radicular cyst was given.

Investigations

**Figure 2:**
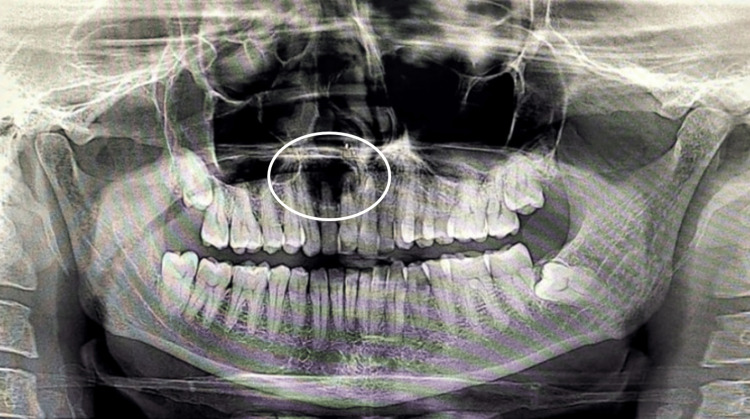
Preoperative OPG Showing periapical radiolucency in relation to 11,12 OPG: Orthopantogram.

**Figure 3:**
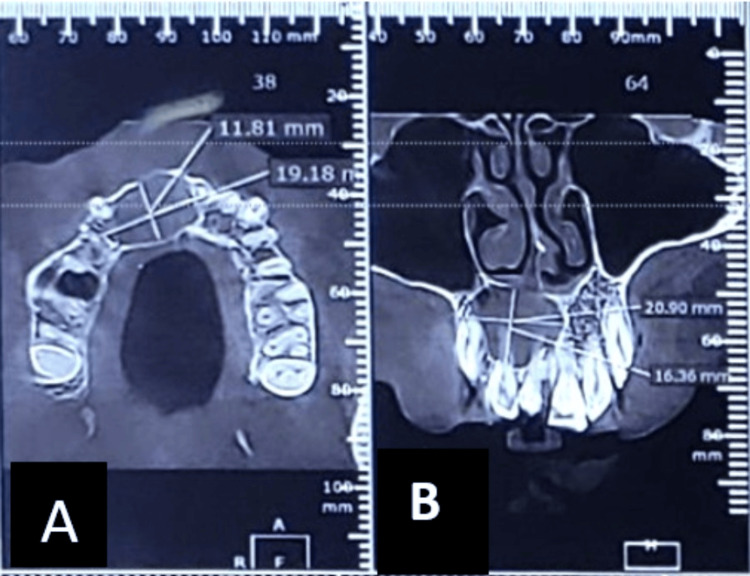
Preoperative CBCT (A) Axial and (B) coronal planes showing expansion of labial and palatal cortical plates. CBCT: Cone beam computed tomography.

**Figure 4:**
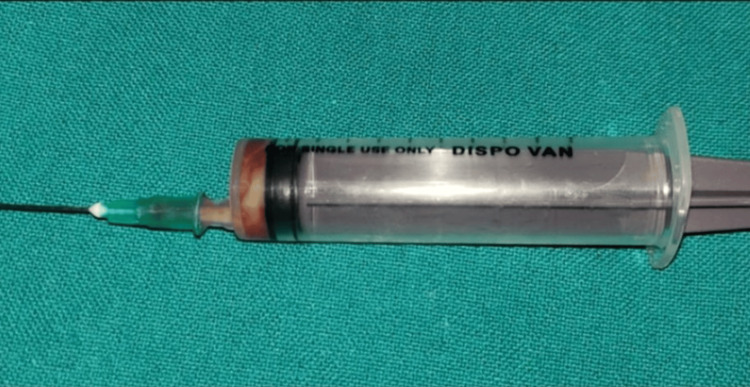
Aspiration output: pus and blood

Initially, an orthopantogram (OPG) was taken to determine the extent of the lesion, which revealed a lesion involving the periapical region of 11, 12 ( Figure 2). Later, cone beam computed tomography (CBCT) was performed, which revealed a Hypodensity measuring about 11.81 mm x 16.36 mm mesio-distally on axial section, 20.90 mm x 16.36 mm labio-palatally on coronal section, causing expansion of labial and palatal cortical plates (Figure 3). On aspiration, there was discharge containing pus and blood (Figure 4).

After a comprehensive clinical and radiographic assessment, the treatment plan was made, which included complete enucleation of the cyst with curettage, followed by apicectomy of teeth 11 and 12 with retrograde filling, and placement of a curcuma oral gel curenext® (Abbott Healthcare Pvt. Ltd., Thane, India)-coated absorbable gelatin sponge Abgel (Healthium Medtech Pvt. Ltd., Mumbai, India) in the surgical cavity. Subsequently, the proposed treatment was explained to the patient in a clear and comprehensible manner, allowing them to fully understand the recommended procedures, potential risks, and expected outcomes, and informed consent was obtained.

Procedure

**Figure 5:**
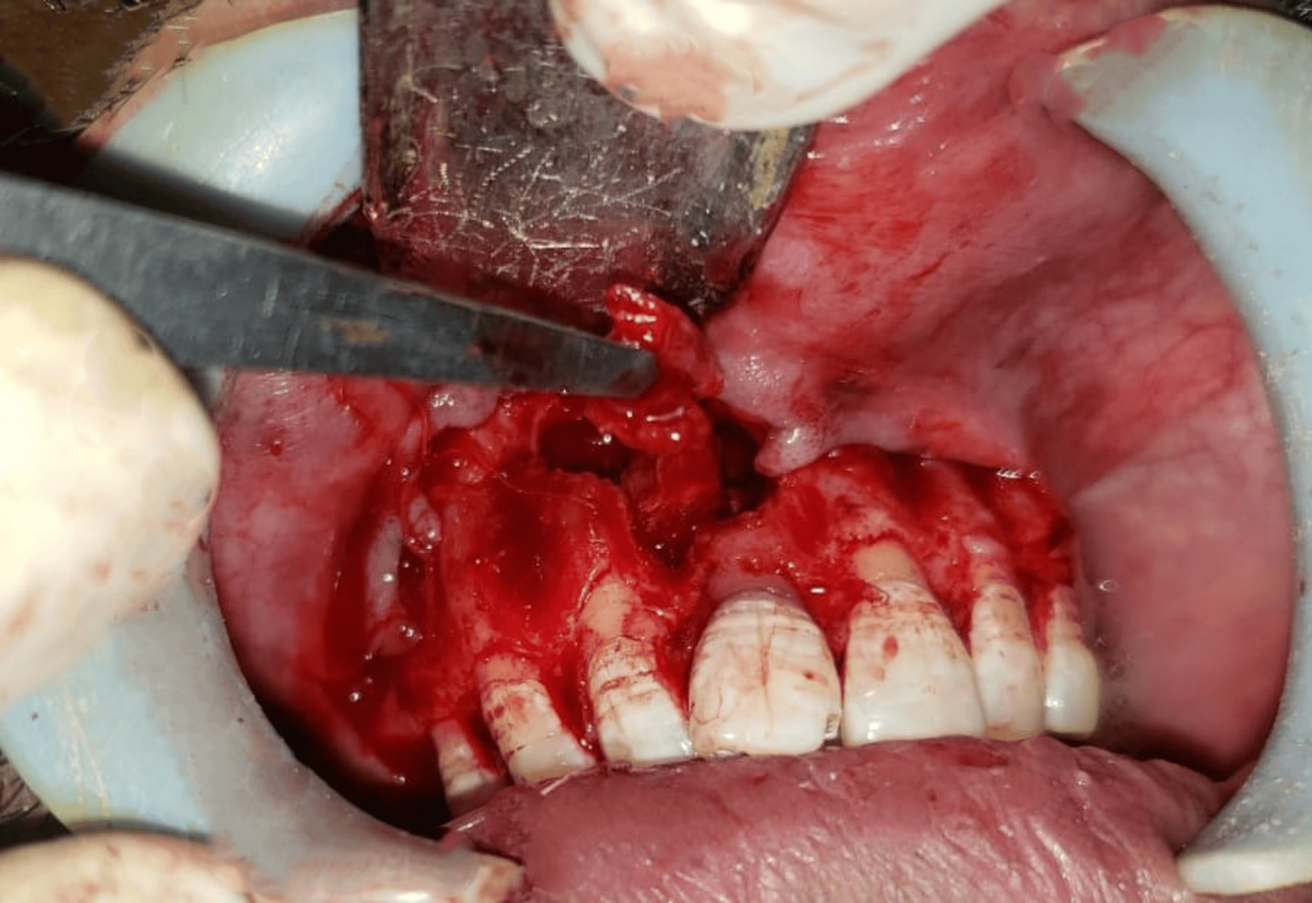
Enucleation of the cyst

**Figure 6:**
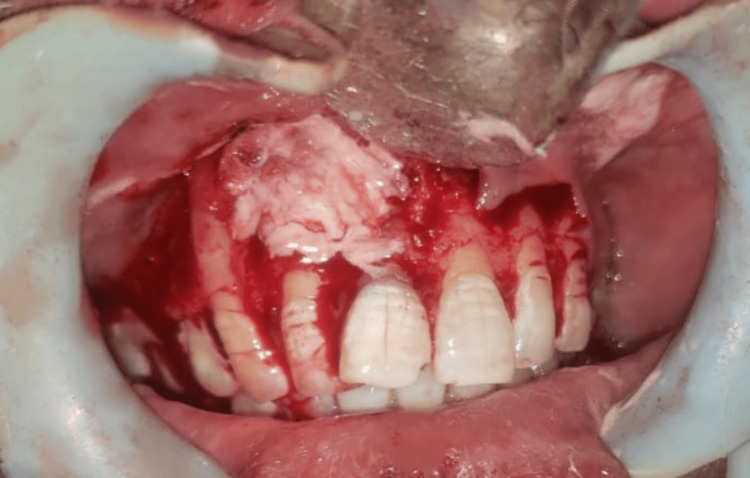
Curcumin gel placement in the cystic cavity

**Figure 7:**
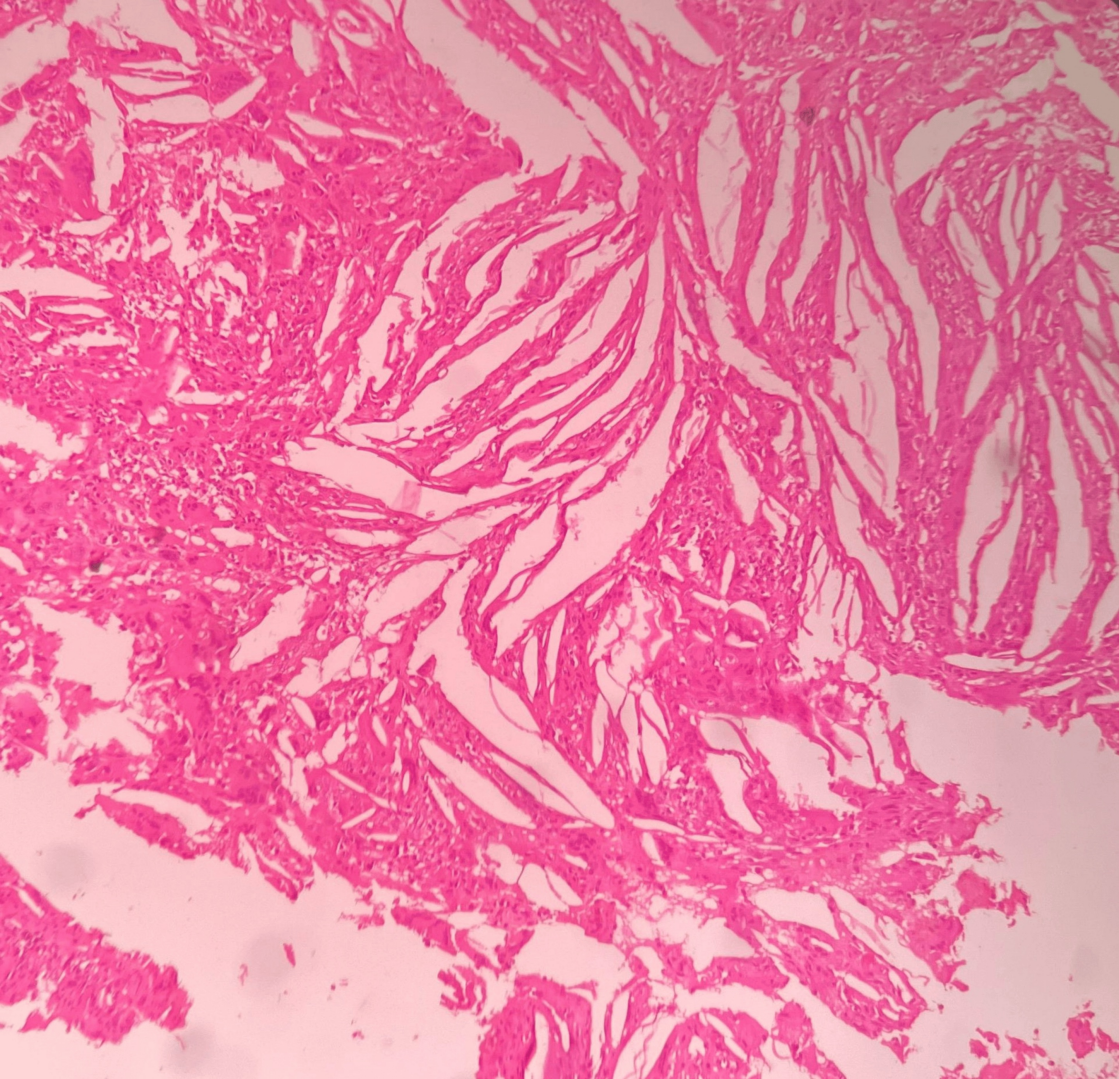
Histopathological examination Epithelium is of varying thickness, showing an arcading pattern, an inflammatory cell infiltrate of lymphocytes and plasma cells.

Under sterile aseptic conditions, 2% lignocaine HCl with 1:80,000 dilution of adrenaline was administered. A crevicular incision was given on the labial aspect extending from distal to 13 to distal to 23, and a full-thickness mucoperiosteal flap was reflected. Complete enucleation of the cystic lesion, followed by thorough curettage, was performed, and the specimen was sent to histopathological examination (Figure 5). Subsequently, apicectomy of 11 and 12 was done along with retrograde filling. Later, absorbable gelatin sponge coated with curcumin gel was placed in the cavity, and the closure was done using non-absorbable 3-0 black braided silk suture material (Figure 6). The patient was prescribed antibiotics and analgesics, and necessary postoperative instructions were given. Histopathological examination reveals that the epithelium was of varying thickness, showing an arcading pattern, inflammatory cell infiltrate of lymphocytes and plasma cells, confirming the diagnosis of infected radicular cyst (Figure 7). The patient was recalled on post-operative one, seven days, and three, six months. 

Outcome and follow-up

**Figure 8:**
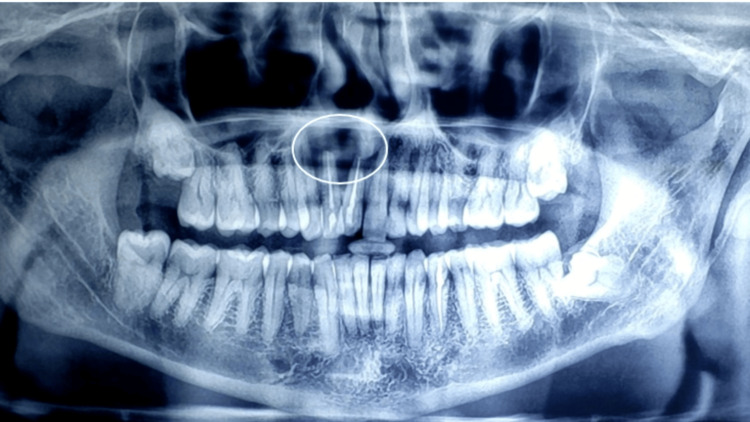
One month postoperative follow up OPG OPG: Orthopantogram.

**Figure 9:**
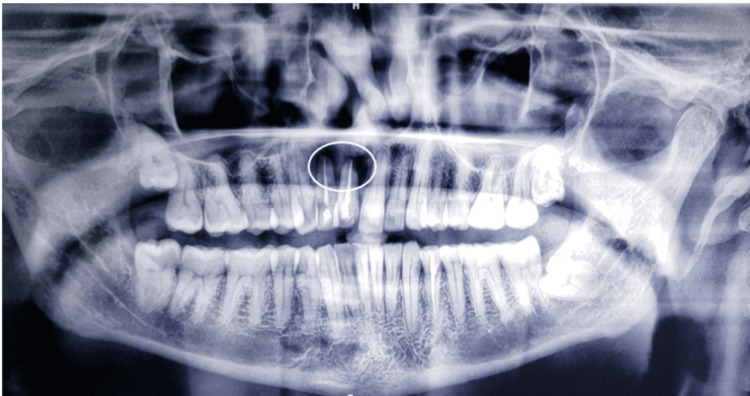
Six months postoperative follow up OPG OPG: Orthopantogram.

On post-surgical follow-up day 1, there was mild to moderate extraoral swelling on the operated side, notably less than what is typically observed following traditional surgical treatment. Pain level as perceived by the patient recorded a visual analogue score (VAS) of 2; intraorally, an absence of erythema (Landry index-Score 4). On postsurgical follow-up day 7, no appreciable extraoral swelling was noticed, VAS-1, Landry index-Score 5, and the intraoral sutures were removed. An OPG was taken after one and six months, which revealed a considerable reduction of periapical radiolucency, and the patient has no other fresh complaints (Figures 8, 9). The patient was further referred to continue endodontic treatment.

## Discussion

A radicular cyst, alternatively referred to as a periapical cyst, typically correlates with a carious, nonvital, discolored, or fractured tooth [9,10]. The formation of the cyst is thought to occur through the proliferation of Malassez's epithelial cell rests in inflamed periradicular tissues [11]. The treatment of these cysts is still a topic of discussion, with many professionals favoring a conservative approach using endodontic techniques. However, in cases of large lesions, endodontic treatment alone may not be sufficient, and it may need to be complemented with decompression, marsupialization, or even complete enucleation [12]. In the present case, surgical enucleation with apicectomy and retrograde filling, followed by placement of gel foam coated with curcumin gel, was performed.

According to Maulina et al. Curcumin is a turmeric-containing active ingredient that has been proven to be effective in treating pain and inflammation due to its analgesic as well as anti-inflammation potential [13]. According to Hussain et al. Curcumin and its derivatives have gained widespread popularity due to their well-documented anti-inflammatory, antioxidant, anti-infective, and antimicrobial activities [14].

Curcumin exerts its anti-inflammatory effects by mitigating the inflammatory response in TNF-α-stimulated human endothelial cells through interference with NF-κB. Additionally, curcumin has the ability to prevent platelet-derived growth factor (PDGF) [15]. The absorbable gelatin sponge functions exceptionally well as a drug carrier [16]. Various dental applications of curcumin, such as its use in mouthwashes, subgingival irrigants, pit and fissure sealants, and as a component in local drug delivery systems in gel form. These applications leverage curcumin's anti-inflammatory and antimicrobial properties to promote oral health [17].

Curcumin has shown promising therapeutic potential in various oral conditions due to its anti-inflammatory, antioxidant, and antimicrobial properties. It is effectively used as an adjunct in periodontal therapy to enhance healing after scaling and root planing, and in managing oral mucositis by reducing pain and inflammation. Curcumin also aids in treating potentially malignant disorders like oral submucous fibrosis and leukoplakia, helping reduce fibrosis and oxidative stress. In oral cancer, it suppresses tumor progression and supports immune response. Additionally, it is beneficial in conditions like recurrent aphthous stomatitis and lichen planus, and serves as a safer alternative to chlorhexidine in maintaining oral hygiene [18].

## Conclusions

In conclusion, the management of a radicular cyst through enucleation with the application of curcumin gel and gel foam represents a novel and promising technique. This case report highlights the potential of curcumin as an adjunctive therapy in enhancing surgical outcomes and facilitating accelerated wound healing with no further complications.
